# Recent Advances in the Mechanisms of Quality Degradation and Control Technologies for Peanut Butter: A Literature Review

**DOI:** 10.3390/foods14010105

**Published:** 2025-01-02

**Authors:** Xinyan Liu, Xuchun Zhu, Zhaowei Han, Hongzhi Liu

**Affiliations:** Key Laboratory of Geriatric Nutrition and Health, Ministry of Education, School of Food and Health, Beijing Technology and Business University, Beijing 100080, China; xinyanofficial@163.com (X.L.); xxcy08@163.com (X.Z.); hanzhaowei2023@163.com (Z.H.)

**Keywords:** peanut butter, nutrient composition, quality deterioration, processing, regulatory techniques

## Abstract

As the quality of life continues to improve globally, there is an increasing demand for nutritious and high-quality food products. Peanut butter, a widely consumed and nutritionally valuable product, must meet stringent quality standards and exhibit excellent stability to satisfy consumer expectations and maintain its competitive position in the market. However, its high fat content, particularly unsaturated fatty acids, makes it highly susceptible to quality deterioration during storage. Key issues such as fat separation, lipid oxidation, and rancidity can significantly compromise its texture, flavor, and aroma, while also reducing its shelf life. Understanding the underlying mechanisms that drive these processes is essential for developing effective preservation strategies. This understanding not only aids food scientists and industry professionals in improving product quality but also enables health-conscious consumers to make informed decisions regarding the selection and storage of peanut butter. Recent research has focused on elucidating the mechanisms responsible for the quality deterioration of peanut butter, with particular attention to the intermolecular interactions among its key components. Current regulatory techniques aimed at improving peanut butter quality encompass raw material selection, advancements in processing technologies, and the incorporation of food additives. Among these innovations, plant protein nanoparticles have garnered significant attention as a promising class of green emulsifiers. These nanoparticles have demonstrated potential for stabilizing peanut butter emulsions, thereby mitigating fat separation and oxidation while aligning with the growing demand for environmentally friendly food production. Despite these advances, challenges remain in optimizing the stability and emulsifying efficiency of plant protein nanoparticles to ensure the long-term quality and stability of peanut butter. Future research should focus on improving the structural properties and functional performance of these nanoparticles to enhance their practical application as emulsifiers. Such efforts could provide valuable theoretical and practical insights into the development of stable, high-quality peanut butter, ultimately advancing the field of food science and technology.

## 1. Introduction

**Figure 1:**
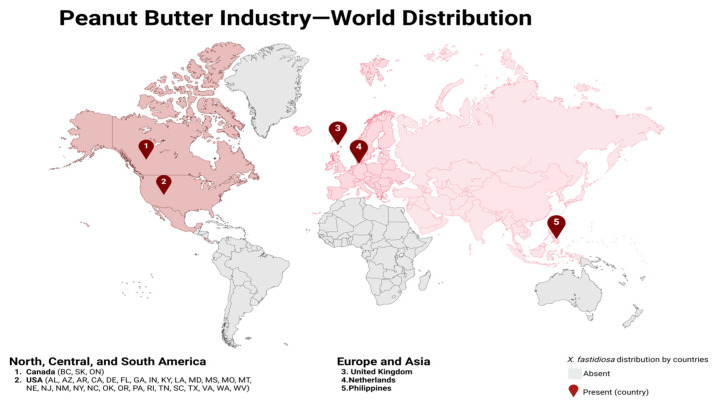
Countries with high consumption of peanut butter worldwide. Source: the figure was drawn using BioRender (https://www.biorender.com/).

Peanuts are the fourth most widely grown edible oilseed in the world and are a good source of protein, fat, carbohydrates, and other micronutrients [1]. Global peanut production varies significantly across regions, with major producing countries including the United States and Brazil. According to data from the USDA (United States Department of Agriculture), peanut production has been increasing year by year from 2000 to 2024. As of 2024, the United States produces over 2.5 million tons of peanuts, with more than 500,000 tons allocated for export. Brazil produces 900,000 tons of peanuts, with 420,000 tons used for export [2]. Peanuts are processed into peanut butter through various techniques to meet the dietary needs of different cultures. For example, in the United States, more than 50% of peanuts are used in the production of peanut butter, which has become an integral part of its food culture [3]. Due to the high nutritional content of peanuts, many developing and underdeveloped countries use peanuts in their daily diet to prevent malnutrition. In general, 53% of peanuts are widely used in the production of edible oils, while 32% are used in food processing (Figure 1) [4].

**Figure 2:**
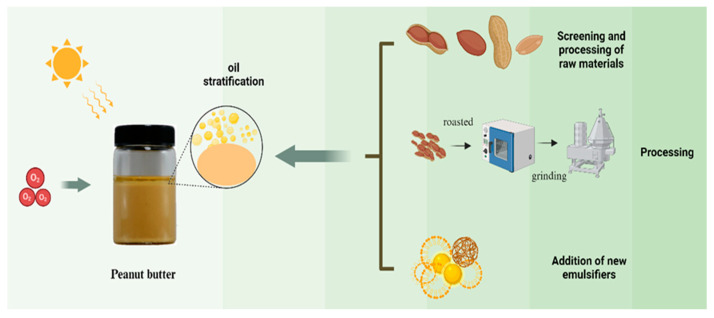
Peanut grease layering (a phenomenon that occurs when oil and sauce are separated) (photo by Xinyan Liu). Source: the figure was drawn using BioRender.

Peanut butter is rich in proteins, vitamins, minerals, etc., and its nutritional richness and unique flavor make it a good meal accompaniment and seasoning product, which is loved by the majority of consumers [5]. The technology used to assess peanut butter plays a crucial role in ensuring its quality and safety. Modern analytical techniques, such as chromatography, mass spectrometry, and dynamic light scattering (DLS), are widely applied to analyze the key components in peanut butter. These technologies not only help researchers accurately identify changes in the composition of peanut butter but also reveal the interactions between ingredients, providing a theoretical basis for regulating and optimizing its quality. Additionally, as a common food product, the quality of peanut butter is closely linked to food adulteration issues. Non-natural ingredients may be added to alter its texture and flavor. This not only affects the consumer’s food experience but can also pose health risks. Therefore, ensuring the purity and quality of peanut butter ingredients and preventing adulteration is a vital aspect of food safety. In recent years, new types of peanut butter such as low-fat, vitamin-enriched, and high-protein types have emerged in response to changing consumer demands. Good organoleptic quality and stability is an important basis for consumers to buy peanut butter. However, during storage, peanut butter may encounter issues such as oil separation, settling of solids, and oxidative rancidity. These problems directly affect the spreadability, texture, flavor, and overall organoleptic qualities of the product, ultimately diminishing its commercial value and limiting the growth potential of the industry (Figure 2). The improvement of peanut butter body stability mainly involves the selection of raw materials, production process control methods, and the addition of food additives. This literature review examines peanut butter as a representative multiphase food system, with a primary focus on the mechanisms underlying stability degradation and recent advancements in stabilization technologies. This literature review specifically (a) examines how the interactions between key components, such as proteins and fats, impact stability; (b) explores how these interactions influence sensory characteristics like texture, spreadability, and flavor; (c) summarizes stabilization techniques and proposes optimization strategies; and (d) highlights current research directions and potential areas for future exploration. Through this analysis, this review aims to provide a scientific foundation to enhance the quality, nutrition, and shelf life of peanut butter, while advancing product development and storage technologies.

## 2. Peanut Butter Quality and Its Challenges

The organoleptic and physicochemical properties of peanut butter play a crucial role in determining consumer preference and overall product quality. For peanut butter, its aroma and flavor are key factors that influence its popularity. The fresh, nutty aroma and strong flavor of peanuts tend to attract consumers, while separated or rancid oils can significantly impact taste and create a negative impression of the product. In terms of texture, the smoothness, graininess, and consistency of peanut butter are directly related to its ease of use and taste experience. Physical and chemical properties are also critical in ensuring product quality and shelf life. The fat content and the proportion of unsaturated fats determine its stability and antioxidant capacity. Additionally, physicochemical characteristics such as moisture content, pH, and viscosity affect the flow and stability of peanut butter as well as its shelf life. Therefore, balancing sensory and physicochemical properties during storage is an important challenge in the food industry. It ensures that peanut butter meets consumers’ taste preferences while maintaining a long shelf life.

### 2.1. Food Quality

#### 2.1.1. Color

So far, the color of peanut butter has consistently been visually appealing to consumers and serves as a key indicator for them in assessing the product’s quality. Numerous experiments have demonstrated that, in addition to perception, the color of a food can significantly influence the true olfactory experience [6,7,8]. In the case of peanut butter, if color expectations are not met, it is difficult for consumers to gain insight into other nutritional quality aspects [9]. The ideal “medium brown” color of peanut butter is produced during the roasting process through a Meladic reaction, which mainly involves the caramelization mechanism of proteins and sugar [10]. During the roasting of peanuts, sucrose is hydrolyzed by converting enzymes to fructose and glucose, and these reducing sugars are then involved in the browning reaction to produce the desired peanut color [11]. Dhamsaniya, Patel, and Dabhi [12] conducted a scientific sensory evaluation of the color of different peanut butter varieties using the color scale from the standard manual as a benchmark. The results indicated that as long as the color remained consistent with the scale specified in the manual, there was no significant variation. Lu et al. [13] evaluated the color of seven varieties of peanut butter by using a fuzzy mathematical sensory evaluation method, and the results showed that the color of the paste was better and in the “excellent” class when the L* value of the color was at 55 ± 1. Dikkala et al. [14] investigated the effect of pressure cooking on the development of peanut butter fortified with peanut skins and studied its physicochemical, functional, and sensory properties. The results showed that the addition of peanut skins significantly increased the color attributes (L* = 40.73~44.76, a* = 4.35~5.57, b* = 7.83~12.43) and phenolic content. In addition, Sanders et al. [15] assessed the effect of peanut skins on the quality characteristics and consumer acceptance of peanut butter and showed that the addition of peanut skins had a significant effect on the appearance and physical characteristics of the product and that the consumer acceptance of color was greater than the flavor, texture, and overall acceptance of the product. This shows that a certain color range is necessary for the preparation of peanut butter.

#### 2.1.2. Flavoring Substance

For peanut butter, flavor is one of the most important quality evaluations and greatly influences the overall consumer acceptability of peanut butter [16]. The formation of peanut butter flavor is a complex process that depends on several factors: genetic composition and growth conditions of the plant, post-harvest handling, processing, and storage conditions. Among them, the peanut roasting process is the most critical factor in peanut flavor formation, and the Maillard reaction, fat oxidation, and protein degradation [16,17,18,19] occurring in peanut kernels during peanut roasting are the main sources of peanut butter flavor, which can significantly change the flavor profile of peanuts [20]. The extraction methods for the volatile components of peanut butter mainly include headspace analysis, solid-phase extraction, solid-phase microextraction, simultaneous distillation extraction, supercritical fluid extraction, and solvent-assisted flavor evaporation [21]. Among them, headspace solid-phase microextraction and simultaneous distillation extraction have more applications in food products [22]. Liu et al. [20] showed that even when processed under the same conditions, the initial concentration of the characteristic precursors of intense peanut flavor in normal oleic acid peanuts was higher than that in high oleic acid peanuts, resulting in more typical volatile components and stronger specific aromas. Lou Fei et al. [23] used HS-SPME–GC–MS (Headspace Solid-Phase Microextraction coupled with Gas Chromatography–Mass Spectrometry) to identify the volatile flavor substances in two commercially available peanut butters, and the results showed that the volatile components of both samples were mainly pyrazines, aldehydes, furans, pyrroles, ketones, and alcohols. ZHOU Yuke et al. [24] used headspace solid-phase microextraction combined with gas chromatography–mass spectrometry (GC–MS) to isolate and identify flavoring substances in four peanut butter samples. The results showed that 71, 93, 58, and 141 volatile substances were identified in samples A, M, X, and Z, respectively. Among these, hexanal, phenylacetaldehyde, 2,5-dimethyl pyrazine, 2-ethyl-3,6-dimethyl pyrazine, and 2-ethyl-5-methylpyrazine contributed significantly to the overall flavor of the four peanut butters. Sample Z contained the most flavor components and had better flavor quality than the other three peanut butters, with its roasted peanut aroma and apricot flavor being more prominent. As a result, peanut butter is rich in a variety of flavor substances, with pyrazines being the main ones. Volatile compounds such as pyrazines play a crucial role in the characteristic flavor of peanut butter, and these compounds produce nutty and roasted aromas that influence the overall acceptability and preference of peanut butter, which is highly valued by consumers.

#### 2.1.3. Texture

In addition to flavor and color, the texture of peanut butter is a very important attribute [25]. The textural attributes of peanut butter are jointly determined through the interaction of a series of rheological properties and sensory characteristics, of which hardness, cohesion, elasticity, and fluidity are some of the most critical ones [26]. The texture of peanut butter is largely a function of the milling and stabilization process [27]. However, some of the differences in texture and rheological properties of peanut butter subjected to the same milling and stabilization processes have been attributed to differences in the chemical composition and metabolites of peanuts [28]. In general, the total lipid content is negatively correlated with texture and rheological properties, such as hardness [29]. This is primarily because the continuous lipid phase acts as a lubricant within the peanut butter matrix. Reducing the lipid content can increase the viscosity of the peanut butter. Optimal fat content can improve the spreadability, making the peanut butter smoother and less prone to separation or drying out during spreading. Conversely, too low of a fat content results in a dry, rough texture that is difficult to spread evenly, while excessively high fat content can lead to an oily, overly fluid peanut butter, compromising its stability and potentially causing oil separation. Adjusting the ratio of fat to protein is essential for optimizing the texture, stability, and sensory properties of peanut butter. Dhamsaniya, Patel, and Dabhi [12] studied peanut butter made from seven different peanut varieties and found that variations in lipid content significantly affected gelatinization and spreadability. Peanut proteins, which constitute the bulk of the solid phase of the peanut butter matrix, also have a significant effect on the texture and rheological properties of peanut butter. The major proteins in peanuts and peanut butter are peanut proteins (14s) and angiosperm proteins (8S, 2S), of which peanut proteins account for about 63% of the total protein in peanut seeds [30,31,32]. It has been reported that some peanut varieties do not have the 35.5 kDa subunit in the structure of peanut proteins [32]. The protein structure of arachin contains the 35.5 kDa subunit and has higher surface hydrophobicity, which may be due to the relatively high proportion of hydrophobic amino acids on the surface, whereas proteins without hydrophobic amino acids have more disulfide bonds on the surface, and therefore have a more stable structure and thermally stable spherical matrix [32]. These variations are likely to influence how proteins interact with lipids, thereby affecting the rate of oil separation and, ultimately, the texture and rheological properties of peanut butter. Proline (Pro) and cysteine (Cys) have been shown to be significantly and positively correlated with texture properties (such as hardness and cohesiveness) and rheological properties (including yield stress). The relatively high hydrophobicity of proline may contribute to the stability of the protein’s spatial structure, while cysteine may enhance the stability of the protein matrix by forming disulfide bonds. Additionally, sugars are believed to mitigate the thermal unfolding of arachidonin, thereby increasing its hydrophobicity and promoting stronger hydrogen bonding within the protein [32]. In a similar study in 2014, Rozalli, Chin, and Yusof [33] studied the preparation of peanut butter from Chinese and Indian varieties and confirmed that the preparation of peanuts from different varieties of peanuts into peanut butter would have some differences in textural properties. This shows that the quality of peanut butter products can be improved by improving the organizational structure of the paste.

### 2.2. Physical and Chemical Quality

The physical and chemical qualities of peanut butter play a key role in shaping consumer preferences and determining the overall quality of the product. Kehong et al. [34] conducted a multipoint trial of eight different peanut varieties in five regions of China to study the degree of influence of varieties and origin factors on the quality of peanuts, and the results of the study showed that, in addition to dietary fiber, the environmental factors of the origin play a significant role in the protein, fat, moisture, and ash content of peanuts, and the varieties mainly have a significant effect on protein, moisture, and ash content. Elsawy, Alessa, and EI-Kholany [35] studied the preparation of peanut butter using peanut shells and evaluated the physical properties of peanut butter. The results of the study showed a significant decrease in lipid content and a significant increase in total fiber compared to the original peanut butter and thus the quality and shelf life of the product. Gong et al. [36] studied different peanut varieties suitable for processing peanut butter. The selected test materials included HuaYu 20, Fuhua 16, Shanhua 19, Four Grains of Red, Rose Red, and Fuhua 10. They evaluated the organoleptic qualities, physicochemical properties, nutritional content, and stability of the peanut butter. The study results showed that the sensory differences between the varieties were relatively small, with Shanhua 19 receiving the highest overall score. In terms of nutritional content, the average fat and protein levels reached 48.75% and 24.58%, respectively. Xian et al. [37] investigated the effect of irradiation on the physicochemical quality of peanut butter and showed that the fat content of peanut butter remained unchanged after irradiation, the protein content changed slightly, the VE content decreased, and the acid value and peroxide value of peanut butter increased with the extension of the storage time, which contributed to the stability of peanut butter.

### 2.3. Nutrient Composition

Peanuts are rich in protein, fat, and a small amount of polysaccharides, as well as various vitamins such as niacin and vitamin E. They also contain a variety of bioactive compounds, including antioxidants, polyphenols, phytosterols, unsaturated fatty acids, proteins, and dietary fiber [24]. From a nutritional perspective, the nutritional value of peanut butter is equivalent to that of peanuts. Therefore, the nutritional quality of peanut butter made from peanuts of different qualities also varies. This quality significantly influences the processing characteristics of peanut butter. Chun [38] found that if peanut butter is made from raw or roasted peanuts, the nutritional quality remains relatively stable, making the nutritional value of peanut butter comparable to that of peanut kernels. The findings are summarized in Table 1.

#### 2.3.1. Protein Composition

Among the essential amino acids in peanut protein, leucine is the highest (6.67 g/100 g), while methionine and lysine are much lower than those of other amino acids. Thus, tryptophan and methionine are limiting amino acids in peanuts. Lysine is the first limiting amino acid in most grains and cereals while peanuts have relatively high lysine content (3.73 g/100 g), and in the diet of some specific groups of people, peanuts can supplement the insufficient lysine intake and improve the bioavailability of proteins, as shown in Table 2 [39,40].

Colombo et al. [41] identified six major subunits in peanut proteins, categorizing them into hydrophobic and hydrophilic types. They found that the presence of denaturants causes the disulfide bonds in peanut proteins to break, leading to a transition from polypeptides to smaller subunit bands. Additionally, Bhushan and Agarwal [42] conducted a study using reversed-phase high-performance liquid chromatography to analyze peanut globulin, revealing that it primarily consists of six subunits: S1 (66.1 kD), S2 (47.9 kD), S3 (42.7 kD), S4 (38.9 kD), S5 (29.5 kD), and S6 (19.1 kD).

#### 2.3.2. Fat Composition

Peanuts are composed of approximately 50% fat, primarily in the form of triglycerides, which account for about 97.4% of their lipid content [43]. The oil extracted from peanuts is predominantly unsaturated fatty acids, constituting around 80% of the total, with oleic acid (53–72%) and linoleic acid (13–26%) as the main components. Additionally, peanuts contain about 20% saturated fatty acids, including palmitic (7–10%), stearic (2–6%), and arachidic acids (5–7%) [44,45].

#### 2.3.3. Composition of Polysaccharides and Other Nutritionally Relevant Ingredients

Peanuts contain around 20% polysaccharides, including approximately 4% starch, with the remainder comprising soluble and insoluble sugars. Their mineral content is relatively low at 2–3%, featuring higher levels of potassium, phosphorus, and magnesium, while being deficient in calcium, iodine, and iron. Peanuts are notably rich in vitamins, particularly vitamin E (26.3–59.4 mg/100 g), which is 4–10 times more than in other cereals. They also provide vitamin C (5.8 mg/100 g) and various B vitamins (B1, B2, B6), essential for energy metabolism and overall tissue health [46]. Among them, vitamin E has an antioxidant effect, maintains normal capillary permeability, enhances resistance, and improves blood circulation.

## 3. Mechanisms of Quality Deterioration in Peanut Butter

Peanut butter is a complex food product with both solid (proteins, carbohydrates, lignocellulose, ash) and liquid (oil, water) phases, making it a thermally unstable multi-phase system. Peanut butter quality deterioration is a complex process resulting from a combination of several physical, chemical, and biological mechanisms that lead to changes in sensory properties and physicochemical characteristics, as well as possible microbial contamination. Together, they accelerate peanut butter spoilage.

### 3.1. Sensory Deterioration

The quality of peanut butter depends mainly on its organoleptic characteristics, including texture, flavor, and mouthfeel. During the roasting process, the sugars and amino acids in peanuts undergo a Maillard reaction, resulting in the formation of brown pigments that significantly alter the color of peanut butter [47]. Darkening of the color of peanut butter is often seen as a sign that the flavor is not fresh and detracts from its appearance.

**Figure 3:**
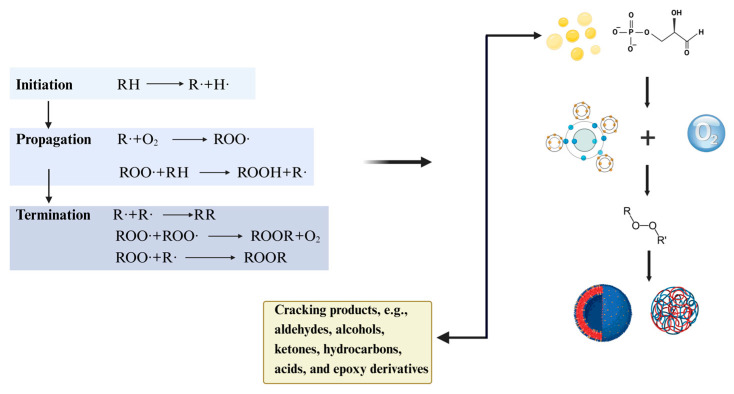
Fat oxidation reaction processes. Source: the figure was drawn using BioRender.

The Maillard reaction, as proposed by Hodge [48], consists of three stages: the primary stage, the intermediate stage, and the advanced stage (Figure 3). In the primary stage, carbonyl–ammonia condensation and rearrangement reactions occur, where the carbonyl group of sugar reacts with the amino group of amino acids to form N-substituted glycosylamines and water, leading to the production of Schiff bases and Amadori or Heyns products [49].

The intermediate stage [50] is crucial for flavor development and involves three reaction pathways: 1,2-enolysis, 2,3-enolysis, and Strecker degradation. During this stage, dicarbonyl compounds react with amino acids, producing carbon dioxide and various aldehydes through Strecker degradation [51]. Changes in color and flavor usually result at this stage.

The final stage [52] is complex, involving reactions such as alcohol–aldehyde condensation, aldehyde–ammonia polymerization, and cyclization. Under the influence of amines, aldehydes and ketones undergo hydroxyaldehyde condensation, leading to nitrogen-free polymerization, rearrangement, and isomerization, ultimately producing melanoidins and other flavor-related compounds [53]. This heating process results in the darkening of the peanut butter’s color, while the generated aldehydes and ketones can impart undesirable flavors, contributing to odor deterioration. Gas chromatography–mass spectrometry (GC–MS) techniques have been employed to analyze the volatile components of peanut butter, identifying oxidation products and other deterioration byproducts such as aldehydes, ketones, alcohols, and acids. The presence and concentration changes of these compounds serve as important indicators of deterioration. Compared to lipid oxidation, the effect of the Maillard reaction on shelf-life is relatively minor, as it mainly affects the organoleptic properties of the food without directly leading to spoilage.

### 3.2. Physical and Chemical Deterioration

The content and physicochemical properties of the raw material components have a significant influence on the stability of the sauce. Peanut butter contains about 50% fat, 25% protein, and 30% carbohydrates and is rich in vitamins and many minerals. Among them, lipid oxidation is the most important non-microbiological cause of peanut butter quality degradation. This process mainly consists of induction, development, and termination phases and induces oxidation reactions by a variety of factors via a complex mechanism, as shown in Figure 3.

Lipid oxidation can seriously affect the shelf life of peanut butter compared to the Maillard reaction. Lipid oxidation significantly affects the shelf life of peanut butter, which has two main stability aspects: the physical stability of the solid and oil phases and the oxidative stability of the oils present. Research has shown that peanut butter made from high-oleic acid peanuts has a significantly longer shelf life than that made from regular peanuts, especially at lower temperatures. This is because high-oleic acid peanuts have a lower linoleic acid content and a higher oleic acid content. Oleic acid, compared to polyunsaturated fatty acids, is more stable and less prone to oxidation reactions [54]. In addition, peanut skins are rich in polyphenols and flavonoids, which are natural antioxidants. The incorporation of peanut skins into peanut butter helps stabilize the emulsion system, thereby extending the shelf life of the product [55,56].

The interaction between lipid molecules and peanut particle surfaces relies on weak van der Waals forces, affecting lipid dispersion and oxidation susceptibility [54]. In peanut butter, peanut particles and fats exist in different physical states, with lipid oxidation primarily occurring in the fat fraction [57]. Since the oils and fats in the solid phase are not fully exposed to oxygen, the oxidation rate is typically slow, and the oxidation products tend to accumulate in the solid phase, impacting the texture and flow properties. These oxidation products may concentrate within the peanut particles, leading to the development of a rancid flavor. In contrast, the fats in the liquid phase are more exposed to oxygen and are more prone to oxidation, often resulting in fat separation, unstable emulsification, or reduced flowability. Additionally, crude proteins, with their hydrophilic and lipophilic groups, stabilize the mixture by balancing polar and non-polar components. Soluble sugars also contribute positively by adsorbing fats and oils, acting similarly to stabilizers. However, the presence of fats and ash can hinder stability due to polar repulsion, negatively impacting quality. Overall, the composition of raw materials plays a vital role in determining peanut butter’s quality.

### 3.3. Microbial Contamination

The degradation of proteins in peanut butter is a complex chemical process influenced by factors such as naturally occurring enzymes and microbial contamination, which can lead to a decline in product quality. Microbial growth and its metabolites—such as volatile fatty acids and amino compounds—often result in off-flavors, typically manifested as rancidity [58]. Microbial contamination is one of the primary factors contributing to the shortened shelf life of peanut butter. The growth of microorganisms not only leads to food spoilage but can also produce harmful toxins, severely compromising food safety [59]. Liquid foods provide a more favorable environment for microbial growth due to their higher moisture content and uniformly distributed nutrients, making microbial contamination in the liquid phase typically more rapid and severe. In contrast, although solid foods may experience localized contamination, microbial growth is generally restricted, resulting in slower changes in flavor and texture. Storage and processing temperatures significantly affect peanut butter quality. Refrigeration helps inhibit microbial growth and slows down enzyme activity, thereby effectively extending the shelf life [60]. Xian Wang et al. [37] demonstrated that treating peanut butter samples with irradiation doses of 0, 1, 3, and 5 kGy significantly reduced microbial counts, with higher doses yielding better results, thereby prolonging shelf life. Changes in pH indicate variations in the acidity of peanut butter, often due to the accumulation of organic acids, which can result from microbial activity [61]. A lower pH is typically associated with increased microbial growth [62]. Peanut butter, being a food rich in fats and proteins, requires strict control of microbial contamination risks during storage. Through appropriate microbial assessment, regular monitoring, and management practices, its shelf life can be effectively extended while ensuring its safety.

### 3.4. Lipid Oxidation, Maillard Reaction, and Microbial Contamination—Interactions

**Figure 4:**
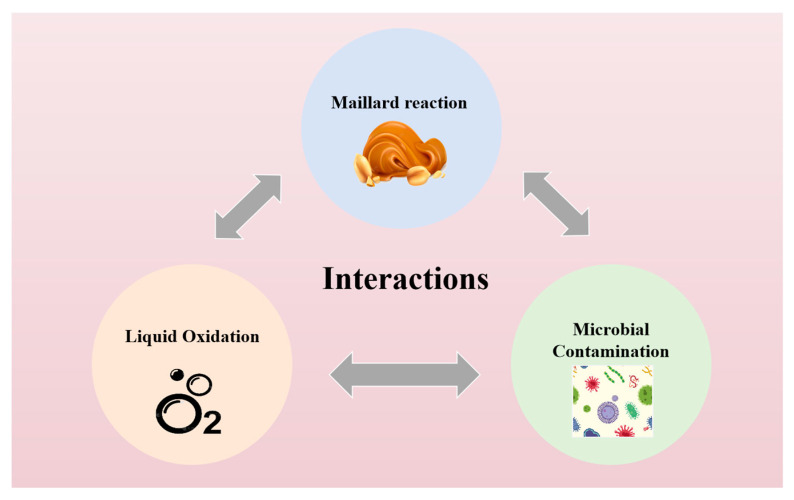
Lipid oxidation, Maillard reaction, and microbial contamination—interactions. Source: the figure was drawn using BioRender.

These processes are interrelated, with a particularly close association between lipid oxidation and microbial contamination, as illustrated in Figure 4. Lipid oxidation not only affects the sensory attributes of peanut butter, but its oxidation products—such as fatty acids and aldehydes—can also serve as essential carbon sources for the growth of specific bacteria and molds. This, in turn, nourishes the microorganisms and accelerates the spoilage process. Furthermore, lipid oxidation can disrupt the structural integrity of the fat molecules and lipid layers in peanut butter, making the surface more porous and facilitating microbial penetration. As microorganisms proliferate in peanut butter, they metabolize and produce acidic substances, volatile organic compounds, and gases, such as ammonia, carbon dioxide, and hydrogen sulfide. These metabolic by-products not only exacerbate the rancid flavors induced by lipid oxidation but also collectively degrade the overall sensory quality of peanut butter, leading to sour and foul odors. Both the Maillard reaction and lipid oxidation contribute to color changes in peanut butter. The Maillard reaction, involving the interaction between sugars and amino acids, results in the formation of brown Maillard products, causing the color of peanut butter to darken over time. Similarly, lipid oxidation can lead to darkening, primarily due to the formation of brown compounds during rancidity. Consequently, during high-temperature storage or processing, the interactions between these two reactions further intensify color darkening, making the peanut butter appear less fresh and adversely affecting its sensory appeal. Additionally, the combined effects of lipid oxidation and the Maillard reaction generate a greater number of volatile organic compounds, which significantly alter the flavor profile of peanut butter. By-products of the Maillard reaction, such as bitter compounds and roasted flavors, interact with lipid oxidation products, including rancid and fishy odors, thereby amplifying the flavor changes in peanut butter. The interaction between the Maillard reaction and microbial contamination is relatively minor, as the Maillard reaction occurs more readily at high temperatures, which also kills some microorganisms [63].

## 4. Techniques for Controlling Quality Deterioration

The processing of peanut butter is vital for its production and quality. Key processes include screening, roasting, milling, and cooling, which can optimize stability and extend shelf life. Understanding the link between processing technology and quality deterioration is essential for both production and consumption.

### 4.1. Screening and Processing of Raw Materials

Initial screening of raw peanuts is crucial for enhancing peanut butter’s stability and delaying quality deterioration. Li Xia et al. [62] found that peanut butter made from high-oleic acid peanuts had lower peroxide and acid values compared to regular peanut butter, indicating better oxidative and storage stability. High-oleic acid peanut varieties have a high content of monounsaturated fatty acids, a fatty acid that is not only beneficial to human health but also enhances the antioxidant capacity of peanuts. Yao et al. [64] examined seven peanut varieties, revealing that those with lower peroxide and acid values also exhibited superior sensory qualities. Gong et al. [36] confirmed that certain peanut varieties, particularly red grains, are more suitable for producing high-quality peanut butter. Furthermore, in addition to unsaturated fatty acids, the moisture content of peanuts directly affects their flavor, texture, and shelf life. Excessive moisture promotes the growth of microorganisms and accelerates the oxidation reaction of peanuts. Therefore, moisture should be strictly controlled before and after roasting, and peanuts are usually required to maintain a moisture content of between 6% and 8%. Physical characteristics such as shell, size, and shape of peanuts also affect processing. Selecting uniform and undamaged peanuts reduces unnecessary losses and ensures overall product quality and taste consistency. It can be seen that the selection of whether the peanut is a good variety of peanut directly determines the quality of peanut butter.

### 4.2. Process Technology Processes

#### 4.2.1. Influence of the Roasting Process on Flavor Composition

Roasting is a critical step in peanut butter production, influencing color, flavor, texture, and shelf life [65]. The interactions between amino acids, reducing sugars, and lipid oxidation products during roasting create the desired flavor and color [66]. Therefore, color and flavor changes during peanut roasting are also accompanied by small changes in the nutritional composition of peanuts [67]. Proper moisture control during roasting is vital, as it affects texture and shelf life. When there is too much moisture, the peanuts cannot be completely dried and are prone to mold and rot; when there is too little moisture, it may lead to over-drying of the peanuts, affecting their taste and nutritional value. Optimal roasting conditions are necessary to prevent bitterness from excessive heat and to ensure a robust aroma. In addition, besides temperature and moisture, roasting time also affects the flavor of peanuts. Proper roasting time helps to release the aroma of peanuts and enhance their nutty flavor. The opposite can lead to a monotonous or even bitter flavor, so the appropriate time needs to be adjusted according to the peanut variety and the roasting equipment. Techniques such as rapid cooling post-grinding can reduce oil separation, while microwave treatment can enhance flavor and stability during storage [68].

#### 4.2.2. Influence of the Grinding Process on Stability

Grinding is essential for refining peanut components and affects the final product’s particle size, viscosity, and overall quality [69]. The milling process typically involves initial grinding followed by homogenization [33]. Longer grinding times yield finer particles, enhancing density and light absorption. The particle size of peanut butter directly influences its texture and stability. Finer particles contribute to a smoother texture, appealing to consumers who prefer a more refined mouthfeel. In contrast, larger particles result in a coarser texture, which can disrupt the balance of the overall sensory experience. Additionally, particle size is related to the oil–water separation in the product. Finer particles tend to promote a more uniform distribution of oils, thereby reducing the occurrence of oil separation. To prevent this issue, it is essential to control the temperature during the grinding process through cooling mechanisms. Typically, maintaining the grinding temperature between 40 °C and 45 °C effectively reduces the occurrence of oxidation reactions, thereby preserving the color, texture, and stability of the peanut butter. However, increased temperatures during grinding can lead to oxidation, impacting flavor and color [70]. Optimizing the fragmentation of peanut cell walls during milling improves both texture and nutrient content, as factors like moisture and temperature significantly affect cell wall integrity [26]. The treatments for milling are shown in Table 3. Optimizing cell wall fragmentation during peanut milling can improve the quality and nutritional value of the final product.

The colloid formed after peanut milling becomes mainly oil and solids with a water content of only about 1%, forming an emulsion suspension of water-in-oil and oil-in-other-substances. When the oil is separated from the solid matrix of the peanut butter, the separated oil may undergo rapid oxidation, and the separation of the oil and solids may alter the rheological properties of the peanut butter, both of which can seriously affect the quality of the peanut butter. The milling process converts the initially discontinuous oil phase dispersed in the solid matrix (carbohydrates, proteins, and other non-fat components) of the peanuts into a continuous oil phase, with the solids constituting a finely dispersed discontinuous phase [26]. The continuous oil phase has a tendency to move out of suspension, resulting in a faster rate of peanut butter deterioration. When peanuts are milled, the degree of dispersion is good, and the system remains relatively stable. However, after some time, stratification occurs. This is because the dispersed particles in the peanut butter are very small, resulting in a large specific surface area and high free enthalpy of the surface, making the system thermally unstable. As a result, fine particles begin to undergo irregular Brownian motion, causing oil molecules to migrate into the oil phase and form small oil droplets. Meanwhile, water molecules move into the aqueous phase, leading to the spontaneous formation of small oil droplets. The small oil droplets spontaneously agglomerate with each other to form larger oil particles. Polymer compounds in the solid phase contain both hydrophilic and lipophilic groups, allowing them to interact with both water and oil. However, the hydrophilicity of these compounds is much stronger than their lipophilicity. As a result, the polymer compounds in the solid phase bind more readily with water than with oil. The presence of water in the sauce causes the small solid particles to absorb water and adhere to one another, forming larger particles. According to Stokes’ law [73], the settling velocity of particles is directly proportional to the particle diameter, directly proportional to the density difference between the two phases, and inversely proportional to the viscosity of the oil phase. The molecules of the solids move towards the solid phase, causing small particles to spontaneously form larger ones. Since the density of the solid particles is greater than that of the oil droplets, the oil droplets float upwards, eventually rising to the top of the sauce and forming a pure oil phase. Meanwhile, the solids sink to the bottom, resulting in the separation of the oil and sauce. In order to reduce oil separation and improve the texture of the product, the following strategies can be used: refinement of particle size; grinding the particles of peanut butter to a very fine size (typically less than 50 microns), which allows the oil to be distributed more uniformly throughout the product and reduces oil separation; and the addition of natural stabilizers, which can increase the consistency of peanut butter and prevent oil separation. Natural stabilizers are not only harmless to health but also improve the stability and texture of the product.

## 5. Food Additive Incorporation Process—Preparation and Application of Plant-Based Protein Nanoparticles

**Figure 5:**
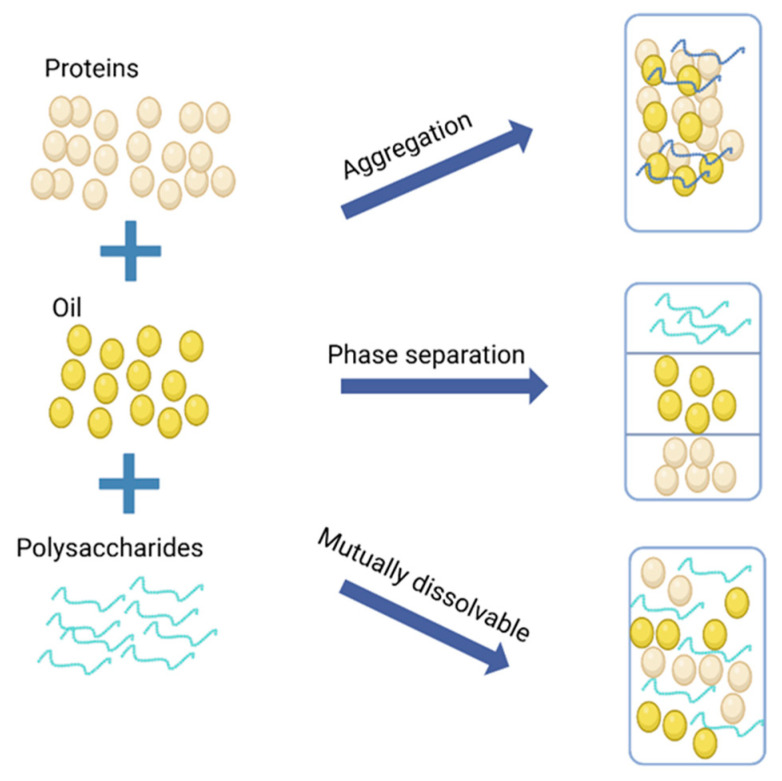
Interaction of peanut butter ingredients. Source: the figure was drawn using BioRender.

In addition to screening, the processing of raw materials and processing technology can regulate the quality of peanut butter deterioration. Adding certain edible additives is also an effective way to improve the stability of peanut butter. Peanut proteins exhibit emulsifying properties, enabling the formation of a stable emulsion by combining the water phase and oil phase. This occurs as proteins interact with fat molecules through their hydrophilic and hydrophobic regions, creating a homogeneous structure that prevents oil and water separation [74]. Additionally, carbohydrates such as cellulose and polysaccharides in peanuts interact with fat molecules to increase the viscosity of peanut butter. This not only improves the texture but also reduces the segregation of oil and fat. The hydration and thickening effects of these carbohydrates are crucial for enhancing the stability of peanut butter [75]. During the preparation process, the interaction between sugars and proteins helps prevent the crystallization or precipitation of fats, further contributing to the overall stability of the product. Currently, food additives to improve the stability of peanut butter involve natural and synthetic products, and the emergence of green food additives has eliminated people’s wariness of food additives in a big way, ensuring food safety and health. In the peanut butter system, protein, carbohydrates, and other components of the colloid exist in a dynamic equilibrium ratio; when the fat component is higher than the equilibrium ratio, it is not conducive to the stability of the system. As shown in Figure 5, if food additives related to substances such as protein and sugar content are added, the content of the substances that will promote the stabilization of the system is increased, and then the stabilization of the sauce can be promoted. Currently, researchers are trying to synthesize protein-based active nanoparticles with high stability by using proteins as carriers for active ingredients [76].

**Figure 6:**
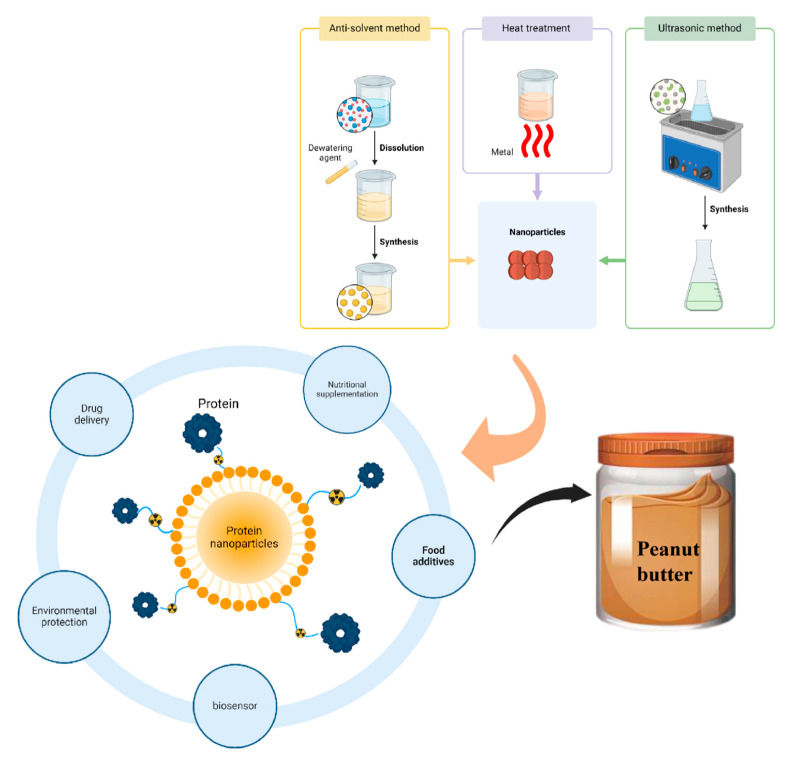
Protein nanoparticle preparation method and the role of plant protein nanoparticles. Source: the figure was drawn using BioRender.

Protein is a favorable material choice for the preparation of nanoparticles because it is an amphiphilic substance containing a high proportion of hydrophobic amino acids, which can generate protein nanoparticles through hydrophobic interactions [77]. The hydrophilic part combines with the water phase to maintain moisture stability, while the hydrophobic part interacts with fat molecules to help form a stable oil–water mixture. This amphiphilic characteristic allows plant protein nanoparticles to effectively prevent oil separation during peanut butter production, maintaining the product’s uniformity and stability. In recent years, the preparation of food-grade nanoparticles using proteins from various plant sources has become increasingly popular among researchers. Due to their small size and large surface area, plant protein nanoparticles can form finer and more stable emulsions in peanut butter. These nanoparticles enhance surface activity, enabling them to effectively combine the oils and water from peanuts, reducing the risk of oil–water separation and improving the texture and appearance of the product. By precisely controlling the size of the nanoparticles (typically within the range of 1–200 nm), their emulsifying capacity, solubility, and bioavailability can be optimized, thereby enhancing food stability and bioavailability. Smaller particles have a larger specific surface area, allowing for better interaction with other components such as fats, carbohydrates, or water, thereby stabilizing emulsions or colloidal systems. Surface modification can impart additional functional properties to plant protein nanoparticles, improving their stability and bioactive delivery capabilities in food applications. Common surface modification techniques include hydrophilic/hydrophobic adjustments, bioactive substance carriers, and edible coatings [78]. Custom plant protein nanoparticles can effectively extend the shelf life of food products. The emulsifying properties of nanoparticles help prevent the separation of food ingredients, such as fat exudation, sedimentation, or crystallization, thereby maintaining the product’s appearance and texture. Furthermore, plant protein nanoparticles can effectively inhibit oxidation reactions, delaying the degradation of fat, vitamins, and other components in food, thus improving food preservation. Custom plant protein nanoparticles also offer significant advantages in bioactive delivery within food. They can protect sensitive nutrients, such as vitamins, minerals, and antioxidants, from degradation caused by heat, light, and oxygen, and facilitate targeted release within the body. Plant-based protein nanoparticles are widely used as emulsifiers and stabilizers in the food industry, e.g., for the preparation of Pickering emulsions or protein gels; they can also be used for loading and protection of bioactives to make them fully utilized [78]. As shown in Figure 6, the main methods for the preparation of plant-based protein nanoparticles include antisolvent precipitation, thermal induction, and ultrasonic methods.

### 5.1. Anti-Solvent Method

The anti-solvent method is the process of forming droplets by dispersing one liquid into another immiscible liquid, also known as liquid–liquid dispersion. In this method, dehydrating agents such as ethanol or acetone are used to remove the hydration film of proteins and reduce the solubility of proteins, and aggregation between protein molecules occurs under the action of self-assembly properties, resulting in the gradual formation of nanoparticles [79]. Other biopolymers are adsorbed on the surface of the nanoparticles, increasing their stability by increasing electrostatic interactions or decreasing hydrophobic interactions [80]. The effect and application of plant protein nanoparticles prepared using the anti-solvent method are shown in Table 4. The anti-solvent method is simple, low-cost, and has a rapid reaction process. It does not require the addition of other surfactants and is suitable for encapsulating various hydrophobic drugs, making it the most widely used method for preparing protein nanoparticles. However, one drawback is that it requires a large amount of ethanol as the solvent phase, which is flammable and poses a risk of explosion [81]. Secondly, it needs to go through a series of cumbersome operations such as solvent evaporation, centrifugation, washing, and drying and is currently limited to laboratory-scale preparation, which is difficult to promote in large-scale applications [82].

### 5.2. Heat Treatment

Heat treatment, as a green process, is a common method for inducing the formation of nanoparticles from proteins. The change in temperature affects the trajectory of protein molecules and thus has a direct effect on the aggregation behavior of proteins. As the temperature increases, it decreases the activation energy of the system, increases the chance of collision between protein molecules, and also increases the rate of movement between molecules, and the increase in temperature also causes changes in the high-level structure of proteins, which leads to the aggregation of molecules and the formation of nanoparticles [89]. The effect and application of using a heat treatment method to prepare plant protein nanoparticles are shown in Table 5. The thermally induced aggregation method is simple and low-cost, suitable for application in an industrial scale, and the exposure of internal hydrophobic groups facilitates better binding to lipophilic bioactives. However, this method relies on the thermal denaturation and aggregation of protein molecules, as well as electrostatic interactions. As a result, the properties of the final composite nanoparticles after heating are influenced by several factors, including biopolymer concentration and ratio, charge density, pH, heating temperature, and ionic strength. In addition, the nanoparticle sizes and shapes of the nanoparticles prepared by this method are not well controlled [90,91].

### 5.3. Ultrasonic Method

The frequency of ultrasound is generally higher than 20 kHz, with frequencies mainly between 20 kHz and 1 MHz [95]. Ultrasound has been widely used in the food industry as a green process [96]. The mechanical and cavitation effects of ultrasound can lead to the dissociation or aggregation of protein subunits and interfere with the non-covalent interactions between natural protein molecules, thus altering the structural and functional properties of proteins [97]. As shown in Table 6, the table illustrates the effects and applications of preparing plant protein nanoparticles using the ultrasonic method. Ultrasonic technology is an environmentally friendly treatment method that requires no chemical additives, offering a high-speed and effective modification process. It has the advantages of being non-toxic, harmless, and preserving nutrients. However, the high-frequency vibrations generated by ultrasound can cause localized temperature increases, which may lead to protein denaturation or degradation, potentially affecting some heat-sensitive proteins.

## 6. Diversified Use of Peanut Butter in the Diet

### 6.1. Improvement of Sleep Problems

Peanut butter is affordable and a great source of monounsaturated fatty acids, which contribute to sleep health. Firefighters’ sleep is often impaired due to the psychological and physical intensity of their jobs and shift schedules [101]. Previous studies have shown that monounsaturated fatty acids may promote sleep health [102]. Firefighters’ dietary patterns include large amounts of red meat and fast food, leading to excessive intake of saturated fat, cholesterol, sugar, and sodium. Therefore, there is a need to identify and promote sustainable dietary choices so that firefighters can easily implement long-term dietary patterns. It has been shown that prolonged exposure to a particular food usually leads to a reduced desire to eat [103]. However, Alper and Matters [104] demonstrated that long-term peanut consumption did not reduce participants’ desire to consume peanuts, thus suggesting that peanuts may be a sustainable dietary addition. Therefore, long-term adherence to peanut consumption appears promising. Oberther et al. [105] used a controlled approach to investigate whether consuming peanut butter at bedtime for 7 weeks improved the quality and/or quantity of sleep, as well as subjective perceptions of mood, attention, and alertness in firefighters located in the southeastern U.S., and the study showed that the food contains natural sleep-enhancing properties.

Similarly, peanut butter has the same effect on astronauts, healthcare workers, and athletes, who work intensely. Peanut butter contains tryptophan, an amino acid that the body uses to produce melatonin, a hormone crucial for regulating the sleep–wake cycle [106]. In everyday life, difficulties in falling asleep or maintaining a restful night’s sleep may result from insufficient production of melatonin by the body. Additionally, magnesium, a mineral found in peanut butter, has been shown to exert a calming effect on the body. Research has established a significant association between magnesium levels and sleep quality, suggesting that adequate magnesium intake may play a crucial role in improving sleep outcomes [106].

For people who work intensely (athletes, firefighters, and paramedics), peanut butter helps provide energy support for extended periods of time, and its rich protein and healthy fats help with recovery and weight maintenance after work. Astronauts, on the other hand, are more concerned with peanut butter’s ease of storage and high energy density. In microgravity, astronauts need an efficient source of energy, and peanut butter is non-perishable, making it ideal for use as food during long-duration space missions [107,108]. In conclusion, peanut butter has demonstrated its broad applicability in a wide range of populations through its high energy density, rich nutrient content, and convenience, especially in the context of the special needs of different populations.

### 6.2. Replenish the Body’s Energy

Peanut butter is rich in protein, which aids in muscle repair and growth [108]. For strength-training athletes, peanut butter is a convenient source of protein, with 7 g of protein per two tablespoons of peanut butter [109]. In addition, peanut butter contains antioxidants, such as vitamin E, which help reduce exercise-induced oxidative stress and protect cellular health [110]. Peanut butter is favored among astronauts and researchers, according to a related report by the National Aeronautics and Space Administration (NASA) [111]. Peanut butter is an energy-dense food containing high levels of healthy fats (especially monounsaturated fatty acids), protein, and dietary fiber, which are important in space missions, especially during long, high-load missions, when astronauts’ energy needs are greatly increased, and peanut butter provides a highly efficient source of energy [112]. Foods in space missions need to not only meet nutritional requirements but also have the ability to be stored for long periods of time without deterioration [113]. Peanut butter has a naturally longer shelf life through its higher fat content and lower moisture content, making it particularly suitable for storage under unrefrigerated conditions. In the microgravity environment of space, the high viscosity properties of peanut butter make it more adaptable [114]. Peanut butter can be an ideal space food precisely because of its good nutrient density, long shelf life, and convenience in a zero-gravity environment. Peanut butter has also demonstrated its unique value in healthcare environments. During cancer treatment, patients may suffer from loss of appetite and weight loss due to chemotherapy or radiotherapy [110].

### 6.3. Preventing Peanut Allergies

**Figure 7:**
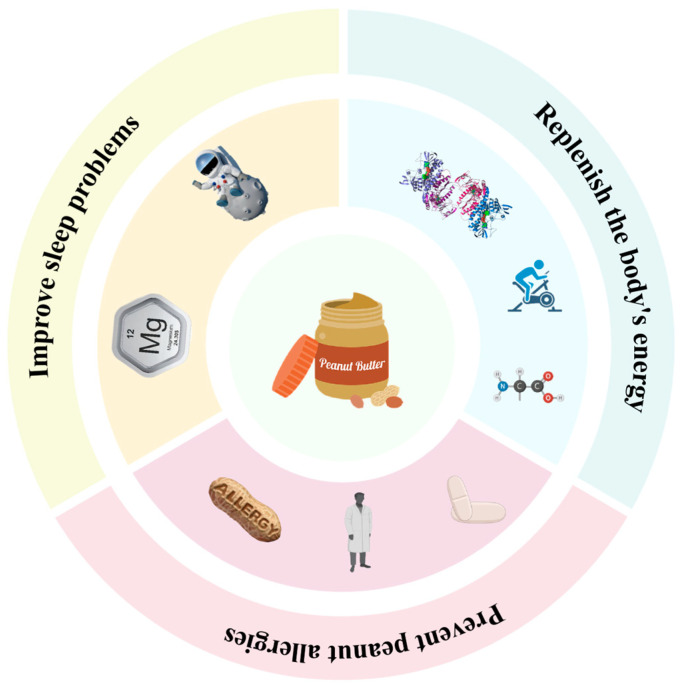
Diversified use of peanut butter in the diet. Source: the figure was drawn using BioRender.

The LEAP (Learning Early About Peanut Allergy) study, a pioneering clinical study of peanut allergy, aimed to explore how a peanut allergy could be prevented through early exposure to peanuts, with findings that early introduction of peanut products (e.g., peanut butter) may help in the regulation of the immune system and reduce the occurrence of peanut allergies [115]. Upon early peanut exposure, the immune system activates regulatory T cells (Treg), which prevent overreaction by modulating the immune response. Thus, peanut exposure drives the immune system toward tolerance rather than an allergic reaction. In addition, peanut exposure reduces the production of IgE antibodies by altering IgE levels in the body, which in turn reduces the occurrence of allergic reactions. The LEAP (Learning Early About Peanut Allergy) research project found that the incidence of peanut allergy was significantly lower in children who consumed peanuts early in life [116]. The appropriate introduction of peanut butter into children’s diets is beneficial due to the immature immune system, as well as the soft texture of peanut butter, which makes it easier for the body to digest and control intake. For patients with established peanut allergies, healthcare professionals need to develop a detailed management plan that includes the identification and avoidance of peanut-containing foods, as well as the use of first-aid medication for anaphylactic reactions when necessary [117]. Diversified use of peanut butter in the diet see Figure 7.

## 7. Conclusions

At present, peanut butter, as a highly processed product of peanuts, is also produced on a relatively large scale. With the expansion of the international market, the peanut butter industry has broad prospects for development. Its nutritional value is rich, almost completely retained from the peanut’s rich vegetable protein, the fatty acid composition of reasonable vegetable oil, and minerals, but it also retains part of vitamin E, sterols, resveratrol, and other natural active ingredients. As a highly nutritious food, the nutritional quality of peanut butter in terms of fat, protein, and vitamin content has been gradually improved, but there are still problems such as separation of oil and grease, short shelf-life, quality degradation, etc., and its development is faced with a number of constraints:

(1) When screening raw peanuts, the equipment may not be able to completely and accurately separate out poor quality peanuts, resulting in an uneven raw material, which can lead to inconsistent flavor or quality issues between peanut butter batches.

(2) Improving the new process may require complex techniques and operations involving precise roasting, grinding, and mixing processes to ensure the taste and texture of the peanut butter. The new process may require recertification of the product and compliance with new regulatory requirements, which can be time-consuming and costly.

(3) The prolonged or excessive consumption of certain stabilizers may not only alter the taste and aroma of peanut butter but also negatively impact human health, which can, in turn, reduce consumer acceptance and sales. This paper reviews various preparation methods and applications of plant protein nanoparticles, highlighting the urgent need to develop a green peanut protein nanoparticle as a stabilizer. Such an innovation could effectively address the issue of peanut butter stratification, providing a sustainable solution for the industry.

(4) The peanut, a prevalent allergen, poses a significant food safety concern that is increasingly prioritized by consumers. The management of peanut allergens is critical throughout the food production process. Effective allergen control typically involves strategies to minimize or eliminate residual peanut proteins during manufacturing. The integration of biotechnology presents substantial potential to enhance product safety, address the growing consumer demand for products with reduced allergy risk, and improve the overall stability of peanut butter.

Future research efforts should prioritize the development of more efficient and naturally derived food additives, the optimization of packaging materials, and the application of advanced biotechnological techniques to enhance both the safety and nutritional value of peanut butter. Moreover, comprehensive, multi-dimensional research is essential, encompassing the selection of peanut cultivars, the refinement of production processes, and the alignment of product characteristics with consumer preferences regarding sensory qualities and health benefits. By promoting integrated research initiatives and fostering technological innovation, these advancements could significantly contribute to improving product quality and facilitating the broader market expansion of peanut butter.

## Figures and Tables

**Table 1:** Nutritional composition (main components) of raw, roasted peanut, and peanut butter.

Nutrient Composition		Raw Peanut	Dry-Roasted Peanuts	Peanut Butter
Basic Nutrient Content/(g/100 g)	Crude Protein	25.80	23.68	25.21
	Crude Fat	49.24	49.66	51.03
	Ash	2.33	3.60	3.25
	Polysaccharide	19.28	21.51	19.28
	Crude Fiber	8.50	8.00	5.90
Mineral Content/ (mg/100 g)	Calcium	92	54	38
	Magnesium	168	176	159
	Phosphorus	376	358	369
	Potassium	705	658	669
Vitamin Content/ (mg/100 g)	Niacin	12.066	13.525	13.403
	Pantothenic Acid	1.767	1.395	0.806
	VE	9.130	7.410	10.000
	Folic Acid	240	145	74

**Table 2:** Analysis of amino acid composition of peanut protein and soy protein (g/100 protein).

Typology	Name	Peanut Protein	Soy Protein	Peanut Protein/Soy Protein Ratio
Hydrophobic Amino Acids	Ala	3.78	4.50	0.84
	Leu	6.67	7.72	0.86
	Ile	2.89	5.02	0.58
	Val	3.68	5.30	0.69
	Phe	5.27	5.00	1.05
	Met	0.91	1.56	0.58
	Pro	3.62	6.20	0.58
	Trp	0.59	1.20	0.49
	Total	27.41	36.50	0.75
Hydrophilic Amino Acids	Ser	5.27	4.61	1.14
	Tyr	4.38	3.91	1.12
	Thr	2.55	3.66	0.70
	Asp	12.61	10.38	1.21
	Cys	2.92	1.63	1.79
	Glu	21.64	18.42	1.17
	Gly	6.21	4.62	1.34
	Total	55.58	47.23	1.18
Sulfur-Containing Amino Acids	Met	0.91	1.56	0.58
	Cys	2.92	1.63	1.79
	Lys	3.73	6.01	0.62
	Trp	0.59	1.20	0.49
	Phe	5.27	5.00	1.05
	Met	0.91	1.56	0.58
	Thr	2.55	3.66	0.70
	Ile	2.89	5.02	0.58
	Leu	6.67	7.72	0.86
	Val	3.68	5.30	0.69
	Total	26.29	35.47	0.74
Others	Lys	3.73	6.01	0.62
	Arg	12.58	7.55	1.67
	His	2.22	2.25	0.99

Note: Those obtained through food include Leucine (Leu), Isoleucine (Ile), Valine (Val), Lysine (Lys), Methionine (Met), Phenylalanine (Phe), Tryptophan (Trp), Threonine (Thr), and Histidine (His) (essential for infants). Non-essential amino acids are those that the human body can synthesize. These include Alanine (Ala), Aspartic acid (Asp), Glutamic acid (Glu), Glycine (Gly), Proline (Pro), Serine (Ser), Tyrosine (Tyr), Arginine (Arg), Cysteine (Cys).

**Table 3:** Treatment and the effect of grinding.

Treatment	Mechanism of Action	Reference
Chemical Treatment	Changing the composition and structure of the cell wall	[71]
Acid–Base Treatment	Partial hydrolysis of the polysaccharide component of the cell wall, making it more fragile and easily fragmented	[71]
Enzymatic Processing	Treatment of peanut cell walls with cellulase or pectinase can significantly improve the efficiency of cell wall fragmentation during the milling process	[72]

**Table 4:** Effectiveness and application of the anti-solvent method for the preparation of plant protein nanoparticles.

Nanoparticle Material	Features/Effects	Appliance	Reference
Glutathione—Sodium Caseinate	Enhanced thermal stability and antioxidant and antitumor properties of curcumin	Curcumin	[83]
Alginate—Chitosan—Zein	Improved photostability, slow release, and bioavailability of resveratrol	EGCG	[84]
Corn Alcohol Protein—Fucoidan Gum	The nanoparticles were prepared with a particle size of 120.8 nm and a PDI value of less than 0.2, which were biocompatible and showed a high encapsulation rate of 95.6% for Ziandra stilbene	Red Sandalwood	[85]
Maize Alcohol Soluble Protein—Pectin	Cinnamon essential oil Pickering lotion has good antibacterial properties	Cinnamon Essential oil	[86]
Carboxymethyl Chitosan—Zein	Enhanced controlled release properties and photochemical stability of vitamin D3	Vitamin D3	[87]
Chitosan—Zein	Enhanced photochemical stability of retinol	Retinol	[88]

**Table 5:** Effectiveness and application of the heat treatment for the preparation of plant protein nanoparticles.

Nanoparticle Material	Features/Effects	Appliance	Reference
Maize Alcohol-Soluble Protein–Sodium Caseinate–Pectin	Nanoparticles exhibit excellent redispersibility and sodium caseinate helps to maintain the original nanosize	Eugenol	[92]
Soy Protein	After adsorption to the interface, it is not easy for structural unfolding and rearrangement to occur, thus maintaining its own intact particle morphology and playing a Pickering stabilization effect	Building Pickering Stabilized Emulsions	[93]
Peanut Protein	Formation of nanoparticles in the particle size range of 176–203 nm; thermal treatment improves the functional properties of peanut proteins	-	[94]
Sodium Arachidonate	Formation of arachidonin nanoparticles in the particle size range of 205–260 nm, which can lead to a significant increase in the anti-coalescence ability of emulsions	Building Pickering Stabilized Emulsions	[86]

**Table 6:** Effectiveness and application of the ultrasonic method for the preparation of plant protein nanoparticles.

Nanoparticle Material	Features/Effects	Appliance	Reference
Soy Protein	Formation of soy protein nanoparticles with maximum emulsion stability	-	[98]
Amaranthine Protein	Ultrasound-assisted mTG caused modification of API nanoparticles that improved particle size, zeta potential, and thermal properties	For Pickering Emulsion Preparation	[99]
Soy Protein	Soy protein nanoparticles with an average particle size of 110 nm were obtained to improve the loading and storage stability of curcumin	Curcumin	[100]

## Data Availability

No new data were created or analyzed in this study. Data sharing is not applicable to this article.
